# Maternal nutrition intervention focused on the adjustment of salt and sugar intake can improve pregnancy outcomes

**DOI:** 10.1002/fsn3.1699

**Published:** 2020-06-10

**Authors:** Yuri Seo, Yeon Seon Jeong, Kyung‐A Koo, Jeong In Yang, Yoo Kyoung Park

**Affiliations:** ^1^ Department of Medical Nutrition Graduate School of East‐West Medical Science Kyung Hee University Yongin Korea; ^2^ Department of Obstetrics and Gynecology Ajou University School of Medicine Suwon Korea

**Keywords:** counseling, maternal nutrition, pregnancy and nutrition, pregnancy outcome, sodium, sugars

## Abstract

Due to the increasing age of pregnant women, maternal nutrition management is becoming more important. Since pregnant women are more likely to consume sodium and sugars than nonpregnant women of the same age, we investigated whether maternal nutrition intervention focused on the adjustment of salt and sugar intake can help pregnancy outcome. This randomized controlled trial was performed on 142 pregnant women within 22 weeks of gestational age for at least 16 weeks until childbirth. Subjects were unequally assigned to the intervention group (*n* = 98) and the control group (*n* = 44). Dietary changes based on perceived taste preferences were evaluated by 24‐hr dietary recall and food frequency questionnaires (FFQ) at pre‐ and postintervention. In the intervention group, while the intakes of energy, protein, and vitamins were maintained, the intakes of sodium (*p* < .001) and sugar from processed food (*p* < .05) were significantly reduced after the intervention. The decreases in salt and sugar consumption were more pronounced in the mothers who had a high preference for saltiness and sweetness. The mean neonatal birth weight of the intervention group was significantly greater than the weight of control group, (3,251.5 ± 402.2 g vs. 2,974.5 ± 294.8 g, *p* < .05). Through this study, nutrition intervention was found to be effective for the formation of healthy eating habits such as reduced salt and sugar intake in pregnant women especially with a high preference for saltiness and sweetness. Also, such specialized maternal nutrition intervention during pregnancy promotes the birth of healthy newborn babies of normal weight.

## Graphical abstract

Nutrition intervention was found to be effective for the formation of healthy eating habits such as reduced salt and sugar intake in pregnant women especially with a high preference for saltiness and sweetness. Also, such specialized maternal nutrition intervention during pregnancy promote the birth of healthy newborn babies of normal weight.

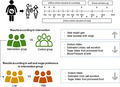

## 1 INTRODUCTION

The maternal diet during pregnancy can play a major role in the mother's reproductive health as well as the infant's growth and health (Abu‐Saad & Fraser, 2010). In recent years, the average age of childbirth has been steadily increasing. The average age of pregnant women in Korea (32.4 years) is the highest among Organization for Economic Co‐operation and Development (OECD) countries, and the proportion of pregnant women over 35 years old has increased to 26.4% (OECD (Organisation for Economic Co‐operation and Development), 2016). This may be partially due to the increasing age of first marriage, rate of remarriage, and number of working women, but this situation has also increased the incidence of maternal pregnancy complications (Centers for Disease Control and Prevention & Health Policy of the Ministry of Health and Welfare, 2017; National Health Insurance Service, 2016). Thus, the nutrition and health care of pregnant women are becoming more important, and nutritional education and counseling are being widely used to improve the health and nutrition of mothers and children.(Girard & Olude, 2012; Nguyen et al., 2017) National studies have demonstrated that interventions related to the intake of macronutrients and micronutrients that are important for pregnant women (such as energy, protein, calcium, iron, folic acid, zinc, and magnesium) have had positive effects, such as preventing pre‐eclampsia, anemia, and nutrient imbalances in babies(Villar et al., 2003). Many healthcare community centers have developed their own programs to provide nutrition education for pregnant women. However, the satisfaction with programs is still low, and only a few studies have reported the effects of nutrition education on maternal outcomes (Kim & Park, 2012). Therefore, it is necessary to study more specialized nutrition education programs and individual counseling to evaluate the effectiveness of nutrition education. In a previous study in 2015, healthcare specialists such as doctors and dietitians performed nutrition education in public health centers. The results demonstrated that periodic nutritional counseling improved pregnancy and neonatal outcomes and facilitated changes in dietary habits (Lee et al., 2017). However, the need for additional research on changes in nutrient intake has been raised, and further interventions related to nutrients that can be overconsumed due to changes in taste during pregnancy (such as salt and sugar) are also required.

Excessive sodium intake is a significant risk factor for cardiovascular disease, cerebrovascular disease, kidney disease, osteoporosis, and gastric cancer (He & MacGregor, 2009; WHO & Consultation, 2003). In 2015, the average daily sodium intake in Koreans from the Korea National Health and Nutrition Examination Survey (KNHANES) was 3,874 mg, which is higher than the recommended intake of 2,000 mg from the World Health Organization (WHO) (Centers for Disease Control and Prevention & Health Policy of the Ministry of Health and Welfare, 2017). Also, in 2012, the percentages of pregnant and lactating women who consumed less than 2,000 mg of sodium per day were 14% and 7%, respectively, which are very low rates. Another study revealed that the increased sodium intake in pregnant women was due to their preference for high‐salt foods, demonstrating the need for nutrition education for pregnant women to control their intake of salt (Im & Cho, 2016). In addition, excessive sugar intake may increase the risk of metabolic diseases such as obesity, diabetes mellitus, and hypertension (Hu, 2013). The 2015–2020 Dietary Guidelines for Americans recommend limiting the intake of added sugar to <10% of total energy and reducing the consumption of added sugar from processed food to less than 25 g (which is 5% of total energy) to help prevent obesity and cardiovascular disease. However, the average daily sugar intake of Korean people is 61.4 g, which represents 12.8% of total energy, and the sugar consumed from processed food is 35 g, which is 7.1% of total energy (Lee et al., 2014). Excessive sweetened beverage consumption during pregnancy reduces the birth of normal‐weight neonates(Günther et al., 2019). On the other hand, reduced sugar intake is associated with reduced blood pressure and blood lipids and the prevention of excessive weight gain (Te Morenga, Howatson, Jones, & Mann, 2014; Te Morenga, Mallard, & Mann, 2012). Considering that pregnant women over 35 years old are more likely to have miscarriages, complications during pregnancy and poor outcomes (Kalewad & Nadkarni, 2017), caution should be exercised regarding sugar consumption according to the trend of the increasing average age of pregnant women. In several studies, the incidence of pregnancy complications such as pre‐eclampsia and gestational hypertension was not associated with sodium intake during pregnancy (Duley & Henderson‐Smart, 1999), and there was no cause‐and‐effect relationship between sugar intake and gestational diabetes mellitus, but excessive sodium and sugar consumption needs to be controlled. The preference for sweetness increases in the mid‐trimester of pregnancy due to changes in taste preferences. The preference for sweetness is the highest and the preference for saltiness greatly increases in the third trimester (Oh & Cho, 2011). Thus, pregnant women in the third trimester prefer salty foods (Im & Cho, 2016) and are more likely to consume sodium and sugars than nonpregnant women of the same age (Cioffi, Figueroa, & Welsh, 2018). Therefore, the nutritional management of salt and sugar consumption should be considered in terms of long‐term health management to prevent chronic diseases related to cardiovascular disease, depending on the characteristics of pregnant women with increased preferences for saltiness and sweetness. However, in previous studies, the restriction of salt consumption in pregnant women also reduced the intake of nutrients that are necessary for the mother and the fetus, such as energy, protein, vitamins, and minerals (van der Maten, 1995). Therefore, when practicing nutrition education, it is important to emphasize the maintenance of major nutrient intakes along with the control of salt and sugar consumption.

The aim of the present study was to investigate the changes in nutrient intake and pregnancy outcomes resulting from nutrition education and counseling focused on the adjustments of salt and sugar intake during pregnancy. In this in‐depth analysis, we compared the nutritional intakes of pregnant women according to their perceived saltiness and sweetness preferences.

## 2 MATERIALS AND METHODS

### 2.1 Study design and population

From March to December 2016, pregnant women aged 20 years old or older and within 22 weeks of gestational age were recruited from three public healthcare centers, five clinics, and one university hospital in the Gyeonggi‐do area. Mothers of multiples and those with metabolic diseases such as diabetes mellitus and hypertension were excluded.

**FIGURE 1:**
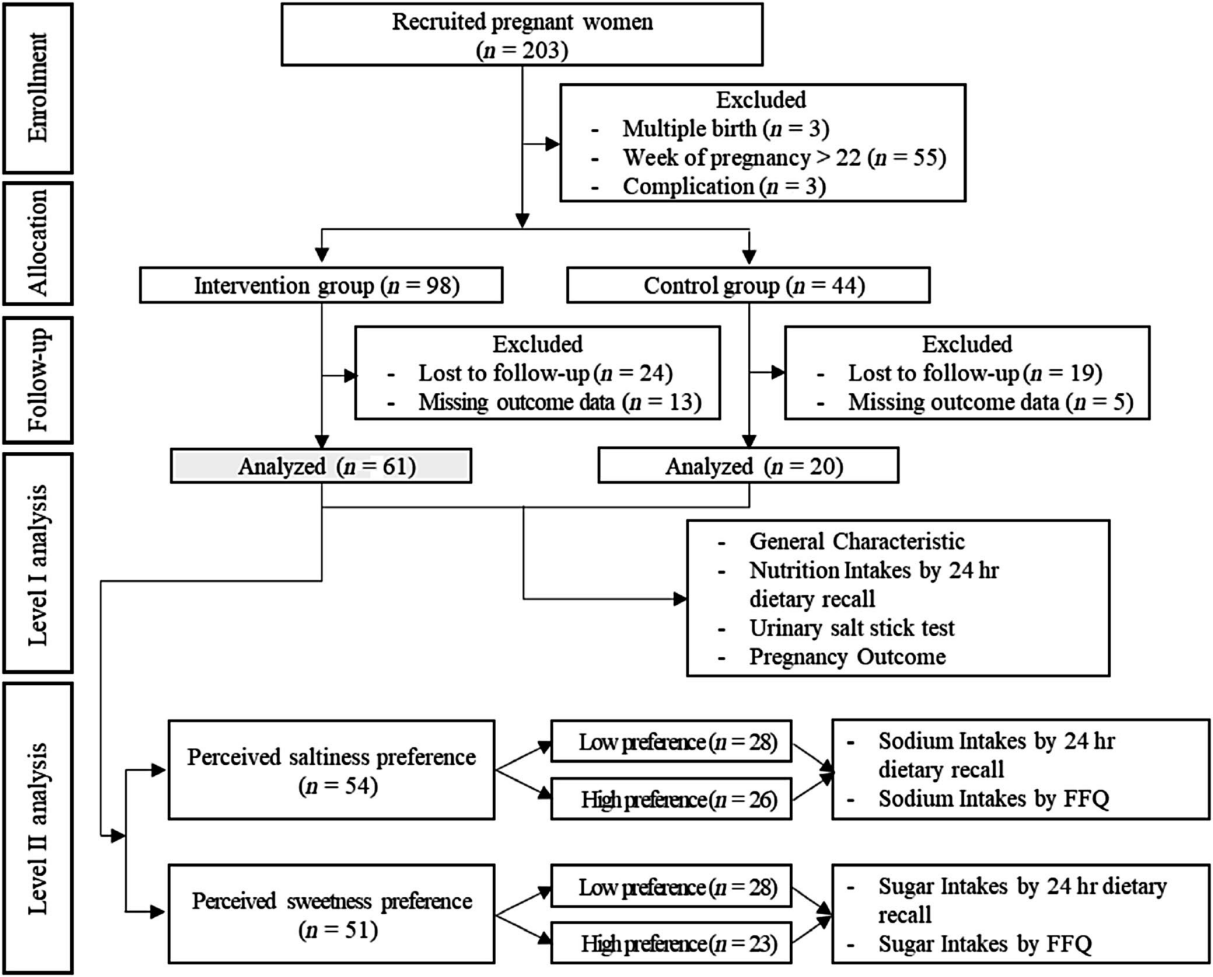
Flow chart of the study population

Initially, 203 pregnant women were recruited to participate voluntarily in the study, but 61 subjects were excluded because they did not meet the selection criteria. After random assignment, subjects were divided into two groups: 98 subjects were assigned to the educational group, and 44 subjects were assigned to the control group. We set the ratio of intervention to control subjects at about 2:1 to provide more education for the target population, minimize the ethical problems related to uneducated subjects, and obtain more information on the effectiveness of the education program. However, during the course of the study, 37 and 24 subjects dropped out of the respective groups due to loss to follow‐up or missing outcome data. The number of subjects was calculated by GPOWER 3.1.9. We used Cohen's guidelines for large (d = 0.8) effect size and α err probability of 0.05, and the power to be 90%. The final total sample size was 78 (Control group = 20), after the ratio of two groups was set to be 3:1 (Cunningham & McCrum‐Gardner, 2007). Ultimately, the results were analyzed in 61 intervention group subjects and 20 control group subjects. Also, in the intervention group, changes in nutrient intake were assessed in 54 subjects divided into two groups according to their perceived saltiness preference, and in 51 subjects divided into two groups according to their perceived sweetness preference (Figure 1).

### 2.2 Nutrition intervention

**FIGURE 2:**
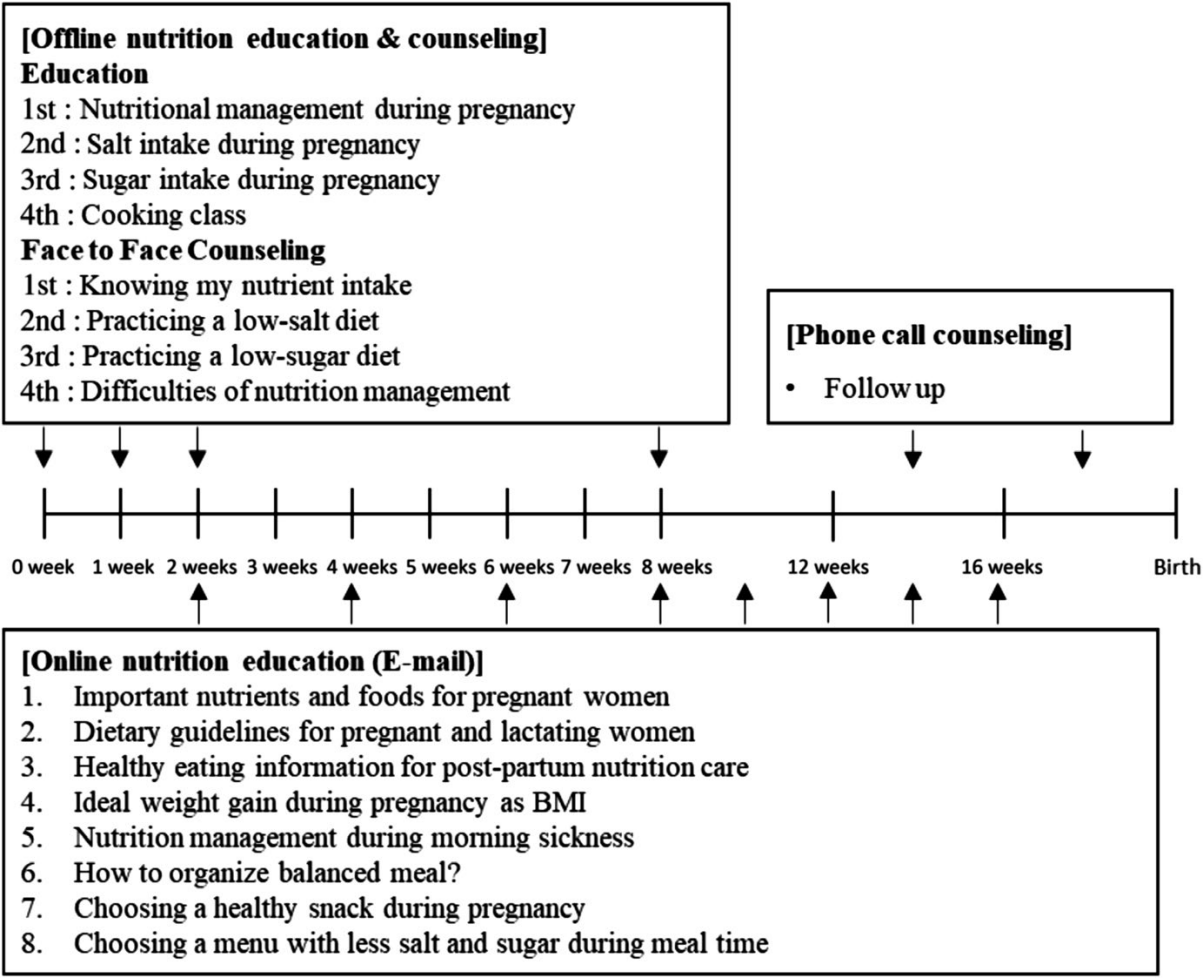
Diagram of the nutrition intervention method

Nutrition education was carried out for 16 weeks by a registered dietitian, and participants were monitored by gynecologists. The content of the education was a diet guideline on pregnant women including practicing method of low‐salt and low‐sugar diet, mainly on educating on the sodium and sugar amount in commercial food products and reducing excessive intake in daily life. The target amount of education for low sugar and low salt on diet was following the World Health Organization (WHO) and “Dietary Reference Intakes for Koreans” (KDRIs) recommendations. During the first 8 weeks of the study, four sessions of offline education and individual counseling were conducted. In addition, online nutrition education with e‐mail education materials was conducted eight times (every 2 weeks for 16 weeks). Follow‐up education was conducted twice before birth via telephone counseling. Subjects in the control group received only 2 times (1st, 4th) offline education (Figure 2).

### 2.3 Measurements

#### 2.3.1 General characteristics

We investigated health‐related characteristics such as the age, gestational age, height, pre‐pregnancy weight, weight at baseline, birth experience, and symptoms that may occur during pregnancy (constipation, anemia, morning sickness, and others) based on participants' self‐reported responses. The systolic blood pressure (SBP) and diastolic blood pressure (DBP) in the stable state at baseline were measured with a blood pressure gauge provided at the public healthcare center. Demographic characteristics including the education level, occupation, household income, family composition, exercise, smoking, nutritional supplement intake (vitamin, iron, folate, etc.), nutrition education experience, and expectations for the program were surveyed through questionnaires.

#### 2.3.2 Nutritional knowledge assessment

The nutritional knowledge assessment was revised and supplemented based on previous research (Kim et al., 2015) and the questionnaire of the National Nutrition^+^ program which is a similar program as WIC(Women, Infant, and Child) program in the U.S. The questionnaire consisted of 13 items, including sodium intake and nutritional management during pregnancy. One point was given if one item was correct, and 0 points were given if the item was incorrect.

#### 2.3.3 Perceived taste preferences

A self‐taste assessment program was used to investigate subjects' preferences for self‐recognition of saltiness and sweetness. First, participants completed a survey on their preferences for and perception of salty and sweet foods. Then, their tastes were determined through the use of 5 different salinity samples (0.08%, 0.16%, 0.31%, 0.63%, 1.25% salt added water) (Shin et al., 2008) and sweetness samples (0%, 2.5%, 5%, 10%, 20% sugar added water) (Park et.al., 2013) provided by the Ministry of Food and Drug Safety. Based on the choice of preference and taste after sampling, the salty taste assessment score was evaluated on a 5‐point scale as “never eating salty,” “do not like salty eating,” “normal,” “sometime eats salty,” or “always eat salty,” and the sweet taste assessment score was evaluated on a 5‐point scale as “never eat sweets,” “do not like eating sweets,” “normal,” “sometimes eat sweets,” or “always eat sweets.”

#### 2.3.4 Dietary assessment

The dietitians investigated subjects' usual intakes by using a 24‐hr dietary recall to record all food items consumed during one day and their amounts. Food models and photographs were used to aid in estimating intakes for each subject. This was conducted two times, at the beginning and the end of the study. The consumption of sodium was roughly determined by means of the Dish‐based Frequency Questionnaire (DFQ 70) (Son, Park, Lim, & Kim, 2007), which estimates sodium intake in Koreans. A revised version of the Food Frequency Questionnaire (FFQ) for sugar intake (Chung, 2007) was used to roughly estimate the consumption of sugar. The validity of the revised FFQ was tested by conducting the test twice in 20–30‐year‐old women about 4 weeks apart. The dietary assessments were analyzed in CAN‐Pro version 4.0 (Computer Aided Nutritional Analysis Program, The Korean Nutrition Society, 2016), and the sugar intake data were analyzed through the database of food and nutrients, the sugar content database (Ministry of Food and Drug Safety), and the nutrition labels of processed foods. The total sugar intake was calculated as the sum of the amounts of sugar consumed from staple foods, milk (cow's milk), fruit and processed food, and then, the processed foods were classified into seven categories: coffee, beverages, snacks, bakery products, soda, dairy products, ice cream, and simple sugar.

#### 2.3.5 Urinary salt estimates

Changes in urinary salt were estimated with the “Self‐Check‐Salt” test kit (JW Medical, Korea) assessed from instantaneous urine of the subjects, considering the limitations of tests performed at public health centers and the convenience of the pregnant women. The estimated urinary salt amount was calculated from the measured values as follows, with the target urine volume set as 200 ml.

[Urine salinity (g) = Urine (ml) × Check salt (g/L) × 1/1000]

#### 2.3.6 Pregnancy outcomes

Pregnancy outcomes were collected from the medical records of the maternal hospital. The maternal outcome variables were the maternal age, weight at delivery, systolic blood pressure (SBP), and diastolic blood pressure (DBP) at delivery, delivery method, and complications during pregnancy. The neonatal outcomes included sex, height at birth, and weight at birth. We also collected the 1‐min and 5‐min Apgar scores, which quickly evaluate the health status of a newborn infant by measuring skin color, pulse frequency, response to stimuli, muscle strength, and respiratory function.

#### 2.3.7 Statistical analysis

The data were analyzed with the Statistical Package for the Social Sciences (SPSS) version 23.0 (IBM). The normality of the data was examined through the Kolmogorov–Smirnov test. The chi‐square test (*χ* ^2^) and Fisher's exact test were used to evaluate the homogeneity of the baseline data for the general characteristics and the pregnancy outcomes of the two groups, and the data were presented as the frequency (N) and percent (%). According to their normality, continuous data were analyzed by an independent *t* test or the Mann–Whitney *U* test and were presented as the mean ± standard deviation (*SD*). Cluster analysis was used to divide subjects into two groups according to the similarity of their taste assessment scores. Differences in average nutrient intakes and urinary salt levels pre‐ and postintervention were tested with a paired *t* test or Wilcoxon signed‐rank test according to the normality of the data. All data were considered significant at *p* < .05.

## 3 RESULTS

### 3.1 General characteristics

There were no significant differences between the control and intervention groups in terms of age, the percentage of subjects over 35 years old, the percentage of primiparous women, height, weight, BMI, blood pressure, or self‐reported clinical symptoms (Table 1). Regarding demographic characteristics, there were no statistically significant differences in educational level, household income, or exercise frequency between the two groups. Also, the intakes of supplementary nutrients such as vitamins, iron, and folic acid did not differ significantly (Table 2).

**TABLE 1:** Health‐related characteristics of the subjects

	Intervention group	Control group	*p*‐value
	(*n* = 61)	(*n* = 20)	
Age (years)	33.2 ± 3.7^a^	33.5 ± 3.6	.751
<35 [*n* (%)]	37 (60.7)^b^	12 (60.0)	1.000
≥35 [*n* (%)]	24 (39.3)	8 (40.0)	
Primiparous [*n* (%)]	41 (62.7)	15 (75.0)	.587
Gestational age (weeks)	14.3 ± 4.6	16.4 ± 4.6	.06
Anthropometric data			
Height (cm)	161.9 ± 5.4	162.3 ± 3.8	.754
Weight at baseline (kg)	60.4 ± 9.5	59.8 ± 11.7	.456
Pregravid weight (kg)	58.3 ± 9.4	57.1 ± 12.2	.259
Pregravid BMI (kg/m^2^)	22.3 ± 3.5	21.7 ± 4.5	.128
<18.5 [*n* (%)]	5 (8.2)	4 (20.0)	.302
18.5–22.9 [*n* (%)]	37 (60.7)	12 (60.0)	
≥23 [*n* (%)]	19 (31.1)	4 (20.0)	
Blood pressure			
Systolic BP (mmHg)	111.6 ± 12.1	112.5 ± 9.6	.595
Diastolic BP (mmHg)	67.4 ± 9.3	70.3 ± 11.1	.209
Self‐reported clinical symptoms			
Anemia [*n* (%)]	15 (24.6)	4 (20.0)	.770
Morning sickness [*n* (%)]	45 (73.8)	16 (80.0)	.767
Constipation [*n* (%)]	20 (32.8)	8 (40.0)	.595

Note

No significant difference between the two groups by the independent *t* test or Mann–Whitney U test at *p* < .05. No significant difference between the two groups by the chi‐square test or Fisher's exact test at *p* < .05.

Abbreviations: BMI, Body mass index; BP, Blood pressure.

^a^ Values are mean ± *SD*.

^b^ Values are *n* (%).

**TABLE 2:** Demographic characteristics of the subjects

	Intervention Group	Control Group	*p*‐value
	(*n* = 61)	(*n* = 20)	
Education [*n* (%)]			
≤High school	2 (3.3)^a^	2 (10.0)	.254
University and above	59 (96.7)	18 (90.0)	
Work status [*n* (%)]			
Not working	38 (62.3)	6 (30.0)	.000***
Working (Full‐time)	1 (1.6)	7 (35.0)	
Working (Part‐time)	4 (6.6)	3 (15.0)	
Maternity leave	18 (29.5)	4 (20.0)	
Household income [*n* (%)] (10,000 won)			
<300	18 (29.5)	8 (40)	.610
301–400	14 (23)	3 (15)	
>400	29 (47.5)	9 (45)	
Exercise [*n* (%)]			
Every day	8 (13.1)	1 (5.0)	.583
1–2 times/week	21 (34.4)	7 (35.0)	
3–4 times/week	16 (26.2)	4 (20.0)	
Sedentary	16 (26.2)	8 (40.0)	
Nutrition supplement consumption [*n* (%)]^b^	60 (98.4)	20 (100.0)	.753
Multi‐vitamin	17 (27.9)	6 (30.0)	.532
Iron	18 (29.5)	9 (45.0)	.275
Folate	41 (67.2)	14 (70.0)	1.000
Others	18 (29.5)	4 (20.0)	.565
Nutritional Knowledge score			
≤10 [*n* (%)]	12 (19.7)	5 (25.0)	.752
>10 [*n* (%)]	49 (80.3)	15 (75.0)	
Nutrition education experience [*n* (%)]	12 (19.7)	7 (35.0)	.223

^a^ Values are *n* (%).

^b^ Multiple responses.

*** Significantly different between the two groups by Fisher's exact test at *p* < .001

### 3.2 Pregnancy outcomes

The mean gestational age at delivery and maternal weight at delivery did not differ significantly between the two groups. The systolic and diastolic blood pressure levels were significantly higher in the control group than in the education group (*p* < .05). There was no significant difference in the chi‐square test for the prevalence of pregnancy complications between the two groups. In terms of neonatal outcomes, the mean birth weight of the neonates was significantly higher in the intervention group than in the control group (*p* < .01). The percentage of newborn infants weighing less than 3,000 g at birth also differed significantly between the two groups (*p* < .05). The Apgar scores did not differ significantly between the groups (Table 3).

**TABLE 3:** Pregnancy outcomes in the two groups

	Intervention group	Control group	*p*‐value
	(*n* = 61)	(*n* = 20)	
Maternal outcomes			
Gestational age at delivery (weeks)	39.4 ± 1.1^a^	38.9 ± 1.1	.165
Maternal weight at delivery (kg)	70.1 ± 9.3	67.7 ± 11.5	.2118
Maternal weight gain (kg)	11.8 ± 5.2	10.6 ± 5.2	.352
Weight gain according to pregravid BMI (kg)			
<18.5	13.0 ± 3.2	10.2 ± 3.1	.286
18.5–22.9	12.7 ± 5.0	11.8 ± 5.6	.640
≥23	9.8 ± 5.6	7.0 ± 5.0	.364
Blood pressure at delivery			
Systolic BP (mmHg)	117.0 ± 9.7	123.9 ± 14.5	.047*
Diastolic BP (mmHg)	71.8 ± 6.5	78.0 ± 12.2	.018*
Mode of delivery [*n* (%)]			
Vaginal delivery	40 (65.6)^b^	21 (55.0)	.432
Cesarean section	11 (34.4)	9 (45.0)	
Complications [*n* (%)]			
Gestational hypertension	0 (0.0)	1 (5.0)	.486
Gestational diabetes mellitus	2 (3.3)	1 (5.0)	
Pre‐eclampsia	0 (0.0)	1 (5.0)	
Others^c^	2 (3.3)	0 (0.0)	
Neonatal outcomes			
Birth weight (g)	3,251.5 ± 402.2	2,974.5 ± 294.8	.006**
Birth weight (<3,000 g)	14 (23.0)	10 (50.0)	.027*
1‐min Apgar score (<7)	1 (1.6)	1 (1.6)	.435
5‐min Apgar score (<7)	1 (5.0)	0 (0.0)	.753

Abbreviations: BMI, Body mass index (kg/m^2^); BP, Blood pressure.

^a^ Values are mean ± *SD*.

^b^ Values are *n* (%).

^c^ Others: Postpartum bleeding, premature rupture of membranes

* Significantly different between the two groups by the independent *t* test or Mann–Whitney *U* test at *p* < .05

** Significantly different between the two groups by the independent *t* test or Mann–Whitney *U* test at *p* < .01

### 3.3 Urinary salt estimates

The mean urinary salt estimate decreased significantly (*p* < .01) in the intervention group, but did not change significantly in the control group.

### 3.4 Dietary intake

The dietary nutrient intakes of the two groups at baseline and postintervention were measured by 24‐hr dietary recall (Table 4). Sodium intake decreased significantly (*p* < .001) in the intervention group. While the total sugar intake of the intervention group did not change significantly during the intervention, the intake of sugar from processed food decreased significantly in this group (*p* < .05). On the other hand, in the control group, sodium intake did not decrease significantly from its baseline value during the study period. Moreover, the intakes of both total sugar and sugar from processed food increased in the control group, although the differences were not significant. In both groups, there was no significant change in energy, carbohydrate, protein, fat, fiber, vitamin, or calcium intake during the intervention.

**TABLE 4:** Dietary intakes at pre‐ and postintervention in the two groups

	Intervention group (*n* = 61)		Control group (*n* = 20)	
	0 weeks	8 weeks	0 weeks	8 weeks
Energy (kcal)	1836.2 ± 434.2^a^	1833.8 ± 405.1	1664.3 ± 304.7	1744.3 ± 436.7
CHO (g)	275.5 ± 65.9	255.2 ± 62.9	240.4 ± 62.5	249.2 ± 68.8
Protein (g)	70.8 ± 23.6	75.2 ± 23.4	67.7 ± 13.9	66.1 ± 23.9
Fat (g)	52.4 ± 21.0	58.4 ± 23.4	48.8 ± 19.0	55.7 ± 20.5
C:P:F (%)	60:15:25	56:16:28	58:16:26	57:15:28
Fiber (g)	21.9 ± 10.1	19.5 ± 6.6	17.4 ± 6.4	17.8 ± 7.6
Vitamin A (μg RAE)	938.1 ± 540.8	876.2 ± 528.8	901.2 ± 395.0	932.4 ± 634.2
Vitamin D (μg)	4.2 ± 6.3	3.2 ± 3.1	4.1 ± 4.2	4.5 ± 7.9
Thiamin (mg)	1.3 ± 0.5	1.5 ± 0.5	2.4 ± 5.8	1.3 ± 0.5
Riboflavin (mg)	1.3 ± 0.6	1.2 ± 0.6	1.2 ± 0.4	1.3 ± 0.6
Niacin (mg NE)	15.5 ± 6.6	15.8 ± 5.8	13.4 ± 3.4	13.7 ± 5.1
Folate (μg DFE)	493.6 ± 217.7	425.8 ± 155.8*	489.4 ± 124.6	452.4 ± 191.6
Vitamin B_6_ (mg)	1.8 ± 0.7	1.8 ± 0.7	1.5 ± 0.6	1.4 ± 0.7
Vitamin B_12_ (μg)	7.4 ± 5.8	6.6 ± 4.9	10.1 ± 16.5	8.2 ± 5.9
Calcium (mg)	587.4 ± 257.7	497.5 ± 214.0	541.7 ± 263.4	540.5 ± 374.9
Iron (mg)	15.8 ± 6.5	13.5 ± 5.0*	19.6 ± 24.0	13.2 ± 5.4
Sodium (mg)	3,891.3 ± 1,374.5	2,751.1 ± 1,080.2***	3,467.2 ± 1,013.9	3,417.9 ± 1665.7
Potassium (mg)	3,048.0 ± 1,329.5	2,864.2 ± 964.2	2,523.4 ± 642.5	2,585.9 ± 1,075.4
Total sugar (g)^b^	56.3 ± 34.1	54.0 ± 25.8	48.8 ± 23.9	60.7 ± 44.6
Sugar from processed food^c^ (g)	18.4 ± 20.3	10.8 ± 12.9*	13.4 ± 15.2	27.0 ± 27.6

Note

Nutrient intakes were measured by the 24‐hr dietary recall method.

Abbreviations: C:P:F, Carbohydrate:Protein:Fat (%); CHO, Carbohydrate.

^a^ Values are mean ± *SD*

^b^ Total sugar: Sugar intake from staple foods, milk, fruit, and processed food.

^c^ Processed food included coffee, beverages, snacks, bakery products, soda, dairy products, ice cream, and simple sugar

* Significantly different between the groups by the paired *t* test or Wilcoxon signed‐rank test at *p* < .05

** Significantly different between the groups by the paired *t* test or Wilcoxon signed‐rank test at *p* < .01

*** Significantly different between the groups by the paired *t* test or Wilcoxon signed‐rank test at *p* < .001

#### 3.4.1 Dietary results in the intervention group based on perceived taste preferences

The dietary intakes of the intervention group were further assessed after dividing participants according to their scores on the perceived saltiness and sweetness preferences (Table 5). The sodium intake at baseline, whether measured by the 24‐hr dietary recall method or the food frequency questionnaire method, was slightly higher in the group with high scores on the salty taste assessment, but the differences were not statistically significant. The total sugar intake at baseline, whether measured by the 24‐hr recall method or the food frequency questionnaire method, was significantly higher (*p* < .01) in the group with high scores on the sweet taste assessment. The sugar intake from processed food at baseline was also significantly higher (*p* < .05) in the high sweet taste assessment score group.

**TABLE 5:** Comparison of dietary sodium and sugar intakes at pre‐ and postintervention based on the perceived taste preferences

	Low‐salt preference (*n* = 28)			High‐salt preference (*n* = 26)		
	0 weeks	8 weeks	*p*‐value	0 weeks	8 weeks	*p*‐value
24‐hr dietary recall						
Na intake (mg)	3,671.0 ± 1,170.5^a^	2,753.9 ± 1,067.9	.005**	3,871.5 ± 1532.6	2,716.8 ± 964.6	.003**
FFQ						
Na intake (mg)	4,403.9 ± 2,147.6	4,074.8 ± 3,449.2	.084	5,422.0 ± 3,094.0	3,412.0 ± 1531.9	.001**

	Low‐sugar preference (*n* = 28)			High‐sugar preference (*n* = 23)		
	0 weeks	8 weeks	*p*‐value	0 weeks	8 weeks	*p*‐value
24‐hr dietary recall						
Total sugar^b^ (g)	41.3 ± 21.5	53.3 ± 30.2	.052	67.8 ± 34.7^1^	57.2 ± 22.7	.141
Sugar from processed‐food^c^ (g)	12.0 ± 14.2	10.5 ± 12.9	.737	25.4 ± 24.7^2^	10.7 ± 13.9	.014*
FFQ						
Total sugar (g)	52.1 ± 28.7	43.4 ± 28.1	.139	83.5 ± 45.6^1^	53.1 ± 39.3	.000***

Abbreviation: FFQ, Food Frequency Questionnaire.

^a^ Values are mean ± *SD*.

^b^ Total sugar: Sugar intake from staple foods, milk, fruit, and processed food.

^c^ Processed food included coffee, beverages, snacks, bakery products, soda, dairy products, ice cream, and simple sugar.

^1^ Significantly different between the groups at 0 weeks by the independent *t* test or Mann–Whitney *U* test at *p* < .05.

^2^ Significantly different between the groups at 0 weeks by the independent *t* test or Mann–Whitney *U* test at *p* < .01.

* Significantly different between the groups by the paired *t* test or Wilcoxon signed‐rank test at *p* < .05.

** Significantly different between the groups by the paired *t* test or Wilcoxon signed‐rank test at *p* < .01.

*** Significantly different between the groups by the paired *t* test and Wilcoxon signed‐rank test at *p* < .001.

In the low‐salt preference group, the sodium intake measured by 24‐hr dietary recall decreased significantly (*p* < .01) during the intervention. However, the sodium intake measured with the food frequency questionnaire did not change significantly. In the high‐salt preference group, the sodium intakes measured by both 24‐hr dietary recall and the food frequency questionnaire decreased significantly (*p* < .01) during the intervention.

In the low‐sugar preference group, sugar intakes measured by all methods did not change significantly during the intervention. In the high‐sugar preference group, the total sugar intake measured by 24‐hr dietary recall did not change significantly. However, the total sugar intake measured by the food frequency questionnaire decreased significantly (*p* < .001). The sugar intake from processed food also decreased significantly (*p* < .05).

## 4 DISCUSSION

In this study, we conducted nutrition education and counseling focused on low‐salt and low‐sugar intake for pregnant women within 22 weeks of gestational age and examined the effects of the education and the factors influencing these effects. The intervention and control groups were analyzed for changes in their dietary nutrient intakes, urinary salt estimates, and pregnancy outcomes. An unequal randomization trial method was used to provide higher levels of nutrition education and counseling to more subjects(Peccei, Blake‐Lamb, Rahilly, Hatoum, & Bryant, 2017), to maximize the clinical effect through education, and to provide more information to the target group(Peckham et al., 2015). Additionally, we analyzed subjects in the intervention group in depth by dividing them into two groups according to their self‐taste assessment scores.

We found that the nutrition intervention increased the percentage of healthy newborn babies of ideal body weight in the intervention group. The mean birth weight was higher in the intervention group than in the control group, and the maternal blood pressure at birth was lower in the intervention group than in the control group. The intervention also had beneficial effects on the diet, as the intakes of sodium and sugar from processed food were significantly reduced.

The mean age of the subjects was 33.2 years in the intervention group and 33.5 years in the control group, and the proportion of women over 35 years old was 39.3% in the intervention group and 40.0% in the control group. In both groups, these values were higher than the Korean averages of 32.4 years and 26.4% (OECD (Organisation for Economic Co‐operation and Development), 2016). It is thought that the age of pregnant women who are interested in education is also increasing due to the increasing average age of pregnant women.

The changes in body weight from baseline to delivery were 11.8 kg in the education group and 10.6 kg in the control group. In the control group, the increase in body weight was less than that recommended by the Institute of Medicine (IOM), which is 11.5–16.0 kg for women of normal BMI. The IOM recommends a weight gain during pregnancy of 12–18 kg for underweight mothers, 11.5–16 kg for normal‐weight mothers, and 7–11.5 kg for overweight mothers. In this study, the average weight gain of the mothers in the intervention group was within the normal range, but the weight gain of underweight and overweight subjects in the control group was less than the recommended amount. As in a previous study, there was a positive correlation between the ideal weight gain during pregnancy and a “balanced diet,” and the nutrition education regarding a balanced diet for pregnant women was thought to have contributed to the ideal weight gain of the subjects(Oh & Cho, 2011). Pregnant women who gain weight within the IOM‐recommended ranges are likely to give birth infant of optimal size(3,000–4,000 g) and are at lower risk for preterm and postmature infants (Olson, 2008). In another study that examined the relationship between the weight gain of the mother and the birth weight of the newborn baby, the percentage of neonates weighing less than 3,000 g at birth was higher among mothers who gained less weight than the IOM recommendation (Rode et al., 2007). In this study, the mean neonatal birth weight was found to be less than 3,000 g in the control group, in which the maternal weight gain was less than the normal range. In contrast, the mean neonatal birth weight of the intervention group was greater than 3,000 g, demonstrating that the nutrition education was effective in promoting a healthy birth weight for the newborn baby.

Compared with “Dietary Reference Intakes for Koreans” (KDRIs), subjects' overall nutrient intake of energy, fiber, folate, calcium, iron, and others (excluding protein and sodium) was as low as about 70%–80% of the recommended intake. The intake of energy was 1,836 kcal in the intervention group and 1,664kcal in the control group which was lower in both groups. This is similar to or higher than the energy intake of 1,635kcal in the 1st‐trimester pregnant women investigated in the recent study (Im & Cho, 2016). The intake of iron and folate was decreased after the intervention, however, the subjects with insufficient intake of iron and folate was not of concern, since the subjects in the intervention group were taking supplements.

In this study, we conducted nutrition education focused on the intakes of sodium and sugar, two nutrients that can be overconsumed due to an increased preference for saltiness and sweetness during pregnancy (Oh & Cho, 2011). At baseline, the intake of sodium was 3,891.3 mg in the education group and 3,467.2 mg in the control group, or about 170% of the 2,000 mg target intake of sodium according to the “Dietary Reference Intakes for Koreans” (KDRIs). These values were lower than the 4,627.7 mg reported for a group of 2,000 pregnant women in 2010, (Centers for Disease Control and Prevention & Health Policy of the Ministry of Health and Welfare, 2017) but the women in this study were still consuming more than the target intake of sodium. A previous study about sodium intake in 197 pregnant women demonstrated that as the gestational age increased, the degree of preference for salinity increased, and the sodium intake was 1.7 times the target intake. This study suggested the need for nutrition education to reduce sodium intake.(Im & Cho, 2016) In the present study, sodium intake was reduced to 2,751.1 mg in the intervention group, while there was no decrease in the control group. The initial sodium intake was similar to that of the previous study, and sodium intake was reduced to 137% of the target intake in the subjects who were educated; thus, “low‐salt intake education” for pregnant women seemed to be effective in reducing sodium intake.

The recommended sugar intake of the KDRIs is 10%–20% of total energy intake, and the World Health Organization (WHO) recommends limiting sugar intake to <10% of total energy intake. They also state that reducing the consumption of added sugar from processed food to less than 25 g, which is 5% of total energy intake, will help prevent obesity and cardiovascular disease (Te Morenga et al., 2012, 2014; WHO & Consultation, 2003). We set the consumption of total sugar at 55 g, which is 10% of the energy requirement for pregnant women aged 30–39 years according to the KDRIs. At the baseline of the study, the total sugar intake was 56.3 g (12.2%) in the intervention group and 48.8 g (11.7%) in the control group, both of which were similar to the standard level of sugar intake, and slightly lower than the total sugar intake of Koreans surveyed in 2014 (61.4 g) (Lee et al., 2014). There was no decrease in total sugar intake (which included sugar from processed food) in either group; however, the intake of sugar from processed food alone was reduced only in the intervention group. While the consumption of sugar from processed food increased about twofold in the control group, it was not statistically significant due to the large standard deviation. In some control group subjects, intake of sugar from processed foods tended to increase sharply due to overconsumption of sweetened beverages, fruit juices, and frequent eating out. A study on taste preferences during pregnancy revealed that the preference for sweetness increased gradually from the second trimester to its highest level in the third trimester (Bowen, 1992; Oh & Cho, 2011), and the most frequently craved foods during pregnancy were found to be sweet foods such as fruits and fruit juices (Bayley, Dye, Jones, DeBono, & Hill, 2002). In our study, as the gestational age increased, the consumption of sugar from processed food decreased in the intervention group but increased in the control group. Therefore, “low‐sugar intake education” for pregnant women helped to promote the proper sugar intake.

Studies designed to reduce the consumption of certain nutrients have limitations in that they generally reduce the consumption of other nutrients such as energy, macronutrients, and micronutrients (Villar et al., 2003). In a previous study in which salt intake was restricted in pregnant women, the intakes of nutrients such as energy, protein, vitamins, and minerals, which are essential for pregnant women, also decreased (van der Maten, 1995). Our study had a very significant strength in that the subjects' salt and sugar intakes decreased after the intervention, while their intakes of energy and macronutrients were fairly maintained.

The self‐reported taste preference for salt has been shown to be able to replace measurements of daily salt intake and 24‐hr urinary sodium levels (Takachi, Ishihara, Iwasaki, Ishii, & Tsugane, 2014). We divided the intervention group subjects into two groups according to their taste assessment scores and compared their changes in intake during the intervention. At baseline, the intake of sodium was lower in the low‐salt preference group than in the high‐salt preference group, and the intakes of total sugar and sugar from processed food were lower in the low‐sugar preference group than in the high‐sugar preference group. As a result of the changes in dietary intake after the intervention, dietary salt intake was reduced only in the high‐salt preference group, indicating that the effect of education was stronger in those who were aware that they were eating salty food regularly and had a high sodium intake. As for the perceived sweetness preference, dietary sugar intake was reduced and the effect of education was stronger in the group with high sugar intake. On the other hand, in the group whose members did not eat sweets, sugar intake was not reduced and normal eating habits were maintained.

However, this study had limitations in that the subjects were a special group as pregnant women which made the study period very long, and there was low participation for various health reasons, the dropout rate was high, and the results of the questionnaires all depended on the subjects' self‐responses. Also, since the study was conducted in a small area, the income distribution was unequal. Besides, most of the subjects who wanted to participate in the research were unemployed or were on leave and it was difficult for employed women to continue to participate in education. Therefore, the dropout rate of such subjects was high in the intervention group and there were differences in the employment status between the groups which makes it difficult to generalize to all pregnant women. We also felt the necessity to develop online or web‐based educational and counseling programs and offer evening or on the weekend program for employed women participate in.

Further, it is difficult to establish the accuracy of the urinary sodium estimates because they were measured with a simple kit. There were also difficulties because the participants in the study were delivered at several hospitals, and the method of recording the results was different in each hospital.

Despite its limitations, this study had several strengths. Unlike general nutritional education studies, this study required subjects to recognize their preferences and eating habits for salty and sweet foods on their own at the time of education, educated subjects on the amounts of sodium and sugar they normally eat, and resulted in desirable changes in sodium and sugar consumption according to the usual eating habits of the subjects.

In conclusion, we suggest providing specialized nutrition education and counseling focused on the adjustment of salt and sugar intake for pregnant women by dietitians during pregnancy was effective in reducing the intakes of sodium and sugar from processed food and in promoting the birth of healthy newborn babies.

## CONFLICT OF INTEREST

The authors declare that there is no conflict of interests.

## ETHICAL STATEMENT

This trial was approved by the Institutional Review Board (IRB) of Ajou University Hospital before the study was initiated. (Approval number: AJIRB‐MED‐SUR‐15–178) Written informed consent was obtained from all study participants before the screening procedure.
